# MLKL in liver parenchymal cells promotes liver cancer in murine metabolic dysfunction-associated steatotic liver disease

**DOI:** 10.1038/s41419-026-08458-x

**Published:** 2026-02-19

**Authors:** Ghiles Imerzoukene, Ghania Hounana Kara-Ali, Céline Heitz-Marchaland, Thibaut Larcher, Mélanie Simoes Eugénio, Annaïg Hamon, Aurore Bidon, Gevorg Ghukasyan, Laurence Dubreil, Nicolas Loiseau, Sarah Dion, Céline Raguenes-Nicol, Claire Piquet-Pellorce, Michel Samson, Marie-Thérèse Dimanche-Boitrel, Jacques Le Seyec

**Affiliations:** 1 Univ Rennes, Inserm, EHESP, IRSET (Institut de recherche en santé, environnement et travail) - UMR_S, 1085 Rennes, France; 2 INRAE Oniris, UMR 703 PAnTher-APEX, Nantes, France; 3 Plateforme d’Histopathologie de Haute Précision (H2P2), Université de Rennes, Rennes, France; 4 Toxalim, Université de Toulouse, INRAE, ENVT, EI-Purpan, Toulouse, France

**Keywords:** Non-alcoholic fatty liver disease, Obesity

## Abstract

The rising prevalence of hepatocellular carcinoma (HCC) in the last decade is mostly attributable to the growing epidemic of metabolic dysfunction-associated steatotic liver diseases (MASLD). However, the transition from steatosis to steatohepatitis (MASH) and ultimately to HCC is not fully understood. As an executioner protein of necroptosis, the mixed-lineage kinase domain-like protein (MLKL) has been proposed to contribute to MASH and HCC development. To investigate its role in disease progression, mice whose liver parenchymal cells (LPCs) no longer expressed MLKL (*Mlkl*^LPC-KO^) were compared to their control counterparts (*Mlkl*^fl/fl^) using an experimental model combining diabetes induction and a high-fat high-sugar diet (HFHSD) for 4, 8, or 12 weeks. Notably, HFHSD failed to induce detectable hepatic necroptosis in *Mlkl*^fl/fl^ mice, with no phosphorylated MLKL observed by Western blot. Both genotypes displayed similar steatosis and mild fibrosis, consistent with comparable MASH severity, and this condition progressed to HCC. Interestingly, the incidence of liver tumors in *Mlkl*^LPC-KO^ mice was significantly reduced, which was associated with a delay in the onset of systemic and hepatic inflammation. At the early stage of the disease (4th week), the absence of MLKL in LPCs appeared to confer a protective effect on the liver, reducing metabolic stress, as reflected by a lower ceramide-to- sphingomyelin ratio, along with oxidative stress and DNA damage. Altogether, our data suggest that MLKL in LPCs contributes to HCC initiation in the context of MASH, potentially involving its described non-canonical role within mitochondria, promoting oxidative stress, a cancer hallmark. This study provides new insights into evaluating the therapeutic potential of targeting MLKL, as its inhibition in LPCs may represent an effective strategy for treating MASH-related HCC.

## Introduction

Primary liver cancers are the third leading cause of cancer-related deaths worldwide, with hepatocellular carcinoma (HCC) accounting for 90% of cases [1]. HCC commonly arises from chronic liver diseases such as metabolic dysfunction-associated steatotic liver disease (MASLD), whose prevalence is rapidly increasing with evolving lifestyles [2]. In the Western world, MASLD will emerge as the predominant cause of HCC in a near future [3]. MASLD is the recently adopted term replacing NAFLD [4] and encompasses the full spectrum of disease, ranging from simple hepatic steatosis to its progressive inflammatory form, the metabolic dysfunction-associated steatohepatitis (MASH, formerly termed NASH). This advanced stage is characterized by hepatocellular injury, liver inflammation, and cell death, with or without fibrosis [5–7]. Lipid overload in hepatocytes triggers lipotoxicity, oxidative stress, and endoplasmic reticulum stress, driving pathological inflammation and hepatocellular damage, thereby promoting progression to MASH. Persistent oxidative stress and inflammation cause DNA damage, fostering oncogenic mutations, hepatic tumorigenesis, and malignant cell expansion [2, 8].

Necroptosis, a lytic and inflammatory form of programmed cell death, has been proposed to occur in human MASH and to contribute to its progression [9, 10]. It involves key effectors like receptor-interacting kinases 1 (RIPK1) and 3 (RIPK3), with mixed-lineage kinase domain-like pseudokinase (MLKL) as the final effector of necroptosis [11]. MLKL disrupts membranes, causing cell lysis and releasing proinflammatory damage-associated molecular patterns (DAMPs). Beyond cell death, MLKL regulates immune responses, inflammation, autophagy, extracellular vesicles, exosomes, and hepatic insulin sensitivity [12, 13]. Several studies using mouse models have reported that MLKL may play a role in the development of MASLD/MASH [14, 15]. Besides, it has been implicated in various cancers, but with dual roles, acting as an anti-tumoral factor (e.g. colon, stomach or ovaries) [16–18] or a pro-tumoral factor (e.g. pancreas) [19]. Recent studies on HCC issued from diverse etiologies have shown that MLKL is more abundant in tumor tissue than in adjacent non-tumor tissue [20]. Moreover, patients with low MLKL levels in tumors had better overall survival. However, its role in MASH-related HCC is still poorly understood. Therefore, our study aims to investigate MLKL’s role in the pathophysiological pathways connecting MASH to HCC. To this end, we followed diabetic mice deficient for MLKL in their liver parenchymal cells (LPCs), which were fed a high-fat high-sugar diet (HFHSD) over a 12-week period. This allowed us to assess the development and progression of MASH to HCC.

## Materials and methods

### Animals and treatment protocols

All animal protocols complied with French laws and institutional welfare guidelines, and were approved by the local ethics committee. License was given by the Ministry of Higher Education and Research (APAFIS#27668-20201013154615_v2). Mice, (C57BL/6 J) were bred in specific pathogen-free conditions in conventional animal facility with 12 h dark-light cycle at 19-20°C. *Mlkl*-floxed mice (*Mlkl*^fl/fl^) [21] were crossed with Alfp-cre transgenics [22] to generate LPCs-specific *Mlkl* knockout mice (*Mlkl*^LPC-KO^). As previously reported [23], these *Mlkl*^LPC-KO^ mice display reduced MLKL expression in the liver, with confirmed knockdown in hepatocyte. Male *Mlkl*^fl/fl^ and *Mlkl*^LPC-KO^ pups received a single 200 µg streptozotocin (STZ) injection (AB142155, Abcam, Amsterdam, Netherlands) at postnatal day 2 to induce diabetes. At weaning (3 weeks of age), diabetic mice were fed *ad libitum* either standard diet (STZ + SD) (Teklad Diet, Envigo, Gannat, France) or HFHSD (STZ + HFHSD) (MD.06414 Adjusted Calories Diet, 60/Fat. Envigo) for 4, 8, or 12 weeks [24].

### Biochemical parameters

Blood glucose was measured at weaning (to eventually exclude non diabetic individuals) and euthanasia using a glucometer (Freestyle Optium Neo, Abbott, Rungis, France). Serum alanine aminotransferase (ALT) levels were determined using the International Federation of Clinical Chemistry and Laboratory Medicine (IFCC) primary reference procedures, with the COBAS PRO c503 Analyser (Roche, Basel, Switzerland). For lipid analysis, liver homogenates were prepared based on total protein content (0.1 mg/mL in ultrapure water; Biosolve, Valkenswaard, Netherlands) using a TissueLyser II (Qiagen, Hilden, Germany). Cholesterol and triglycerides were quantified using enzymatic kits (Boehringer Mannheim, Germany), according to manufacturers’ instructions, and sphingolipids by mass spectrometry as previously described [25]. The coenzyme Q9 (CoQ9) was quantified separately in frozen liver samples homogenized in 2-propanol and centrifuged. Supernatants were analyzed by reverse-phase HPLC with electrochemical detection (Thermo Scientific Dionex 6011RS ultra Analytical Cell) as previously described [26]. Results for lipids were normalized using BCA-assayed protein (Thermo Fisher Scientific, Villebon-sur-Yvette, France), and for CoQ9 using Bradford-assayed protein (Bio-Rad, Marnes-la-Coquette, France).

### Quantitative real-time PCR

Total RNA was extracted from frozen tissue using the NucleoSpin® RNA Kit (Macherey-Nagel, Hoerdt, France). cDNA was synthesized using the High-Capacity cDNA Reverse Transcription Kit (Life Technologies, Courtaboeuf, France). Real-time quantitative PCR was performed using SYBR® Green (Life Technologies) on the CFX384 Touch™ real-time PCR detection system (Biorad, Marnes-La-Coquette, France). Primers are depicted in supplementary data (Table S1). The relative gene expression was normalized to 18S gene expression. Samples from diabetic SD-fed mice of littermate controls were used as reference for mRNA expression (the mean level of control mRNA was arbitrarily set to 1).

### Immunoblotting, histology and immunochemistry

Liver tissues were lysed in RIPA buffer (supplemented with protease and phosphatase inhibitors) using a TissueLyser LT (Qiagen, Germany) and 5 mm stainless steel beads (Qiagen, Germany). After sonication and centrifugation (11,000 × *g*, 5 min, 4 °C), 15 µg of protein were separated by SDS-PAGE, transferred to nitrocellulose, and blocked in 4% BSA/TBS-T. Membranes were incubated overnight at 4 °C with anti-pMLKL (#37333, 1:250, Cell Signaling, The Netherlands) or for 1.5 h at 20 °C with anti-HSC70 (#sc-7298, 1:5000, Santa Cruz, USA), followed by HRP-conjugated secondary antibody. Signals were detected by ECL (Merck, Germany) and imaged using a ChemiDoc XRS+ system (Bio-Rad, USA).

Liver and spleen samples were fixed in 4% formaldehyde at room temperature for 24 h before paraffin embedding. Liver sections (4 µm thick) were stained with Hematoxylin Eosin Safran (HES), or via immunochemistry. A pathologist blindly scored all livers for NAFLD activity score (NAS), preneoplastic lesion severity and for the incidence of adenoma/HCC. Second-harmonic generation (SHG) and two-photon excitation fluorescence (TPEF) microscopy were performed on liver sections using a multiphton microscope A1RMP-HD (Nikon Europe B.V., Amsterdam, Netherlands) equipped with an Insight Deepsee laser (820 nm excitation). Collagen-specific SHG signals were detected in forward combined with circular polarization and quantified using NiS-Elements software (5.40.01, Nikon Instruments Inc., Nikon Europe B.V.), with an algorithm developed to restrict analysis to hepatic parenchyma and exclude perivascular regions. For multiplexed immune histology, paraffin-embedded tissue sections were dried at 58 °C for 1 h, followed by antigen retrieval and incubated with primary antibody using an automated staining machine (Discovery XT, Roche, Meylan, France). The primary antibody was revealed using an HRP-conjugated secondary antibody (Roche) and a DAB substrate kit (Roche). Hematoxylin was used for counterstaining. For fluorescent detection, antigen retrieval and incubation with the chosen primary antibody were conducted on a Roche automated staining machine (Discovery Ultra, Roche). Tyramide signal amplification was applied using Rhodamine, Cy5, FAM or DCC (Roche). An aqua mounting solution containing DAPI was used (SouthernBiotech, Birmingham, USA) for nuclear counterstaining. Images were scanned on Nanozoomer 2.0 RS (Hamamatsu, Massy, France). The protocol describing steps from labeling to image analysis for multiplexed fluorescent immunohistology has already been detailed [27]. Antibodies are depicted in supplementary data (Table S2). For 8-OHdG staining, a QuPath classifier (v0.3.2) was trained to detect 8-OHdG-positive hepatocytes, based on the size of detected objects (nuclei).

### Statistics

Data are presented as means ± SEM. Statistical comparisons were performed only between genotypes at the same treatment time point and their respective SD + STZ controls. Furthermore, normality and equality of variances were assessed, but assumptions for parametric tests were not met. Therefore, mean differences between experimental groups were assessed using the non-parametric Mann–Whitney *U* test in GraphPad Prism8. For contingency tables, Chi-square and Fisher’s exact tests were performed. Each n value corresponds to an independent biological replicate (individual mouse). Dot plot representations were chosen to illustrate data distribution and indicate the sample size (n) for each group; when not applicable, n is provided in the corresponding figure legend. Statistical significances are indicated as: *p < 0.05, **p < 0.01, ***p < 0.001 and ****p < 0.0001 compared *Mlkl*^LPC-KO^ mice to *Mlkl*^fl/fl^ mice under similar experimental conditions; ^$^*p* < 0.05; ^$$^*p* < 0.01 and ^$$$^*p* < 0.001 compared *Mlkl*^fl/fl^ diabetic mice under HFHSD to the *Mlkl*^fl/fl^ control mice under SD; ^#^*p* < 0.05, ^##^*p* < 0.01, ^###^*p* < 0.001 and ^####^*p* < 0.0001 compared *Mlkl*^LPC-KO^ diabetic mice under HFHSD to *Mlkl*^LPC-KO^ control mice under SD.

## Results

### The absence of MLKL in LPCs delayed the intrahepatic inflammatory stage

To investigate the role of MLKL in LPCs during HCC development in the context of MASH, *Mlkl*^fl/fl^ and *Mlkl*^LPC-KO^ mice with diabetes induced by postnatal treatment with streptozotocin (STZ) were subjected to HFHSD feeding for 4, 8, or 12 weeks, a protocol known to induce steatosis, MASH, and HCC [24].

**Fig. 1:**
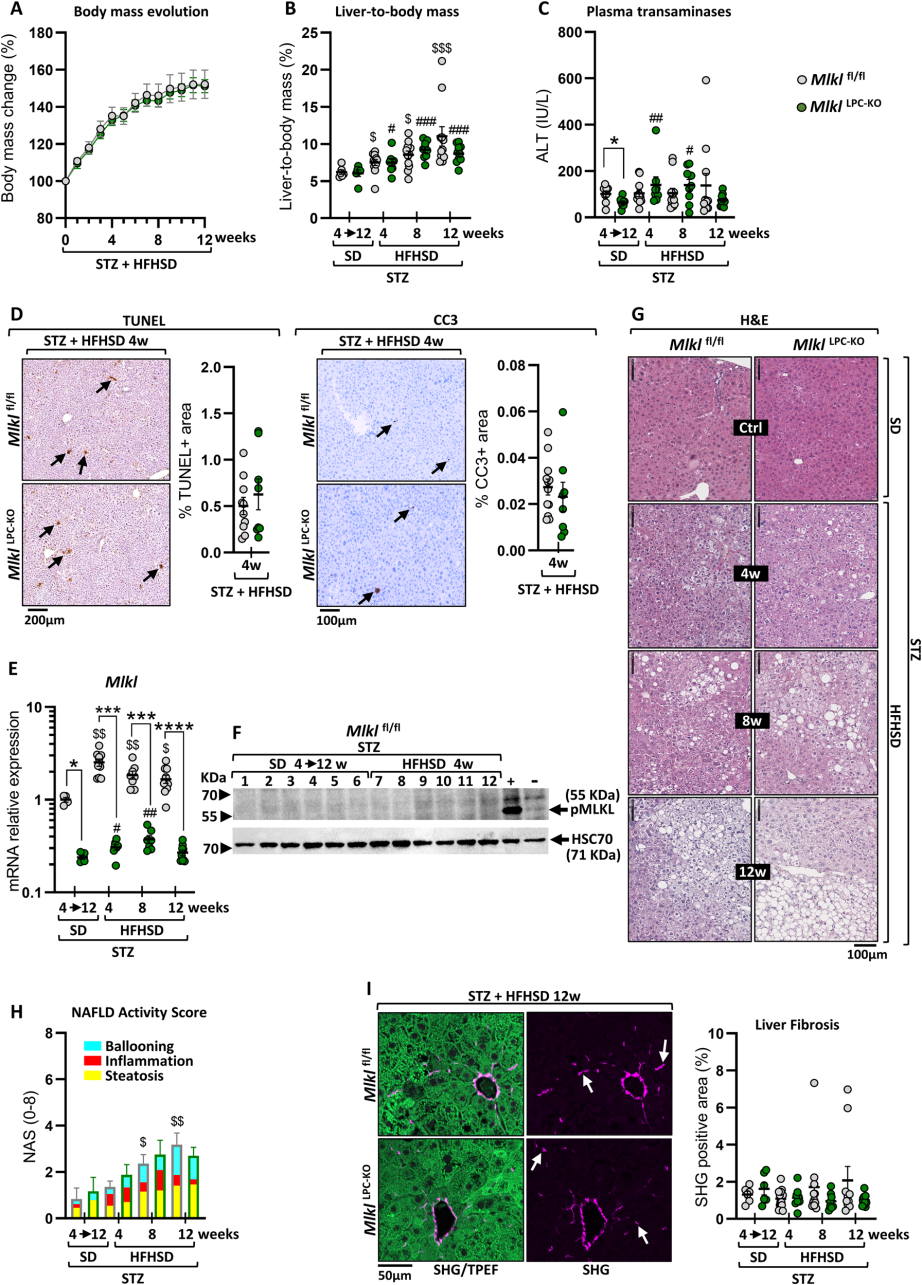
Effect of MLKL deficiency in liver parenchymal cells on MASLD progression. *Mlkl*^fl/fl^ and *Mlkl*^LPC-KO^ mice with streptozotocin (STZ)-induced diabetes were fed either a standard diet (SD) for a period of 4 to 12 weeks or a high-fat high-sugar diet (HFHSD) for 4, 8, or 12 weeks. **A** % of body mass change (n = 12 *Mlkl*^fl/fl^; n = 9 *Mlkl*^LPC-KO^). **B** Liver mass as % of body mass. **C** Serum alanine aminotransferase (ALT) concentrations (IU/L). **D** Representative images of TUNEL staining (left panels) and cleaved caspase-3 (CC3) immunohistochemistry (right panels) on liver sections from *Mlkl*^fl/fl^ (upper panels) and *Mlkl*^LPC-KO^ (lower panels) mice. Arrows indicate positive cells. Scale bars: 200 µm (TUNEL) and 100 µm (CC3). Corresponding quantifications of positive areas are shown next to the images. **E** Hepatic mRNA relative expression of *Mlkl*. **F** Western Blot analysis of phosphorylated MLKL (pMLKL) in liver extracts from *Mlkl*^fl/fl^ mice with STZ-induced diabetes fed either a SD for 4 to 12 weeks (mice 1 to 6) or an HFHSD for 4 weeks (mice 7 to 12). Positive (+) and negative (−) controls correspond to L929 cell lysates treated or not with TNFα/Birinapant/zVAD. Molecular weight markers are indicated on the left. The positions of pMLKL and the loading control HSC70 are shown on the right. **G** Representative pictures of H&E staining of liver sections. Scale bars 100 µm. **H** NAFLD Activity Score (NAS). The NAS score was calculated as the unweighted sum of the scores for steatosis (0–3), lobular inflammation (0–3), and ballooning (0–2), which are represented within the histogram bars in yellow, red, and blue, respectively; sample size (*n*) for each group, from left to right: 6, 6, 11, 8, 11, 8, 11, and 10. **I** Representative images showing merge signals (left panels) of two-photon excitation fluorescence (TPEF; liver tissue autofluorescence, green) and second-harmonic generation microscopy (SHG; collagen, magenta) and SHG signal alone (right panels) from liver sections of *Mlkl*^fl/fl^ (upper panels) and *Mlkl*^LPC-KO^ (lower panels) mice after 12 weeks of HFHSD. White arrows: SHG-positive collagen fibers. Scale bars: 50 µm; corresponding quantifications of SHG-positive parenchymal areas are shown next to the images. Grey and green dots represent *Mlkl*^fl/fl^ and *Mlkl*^LPC-KO^ individuals, respectively. For the chart, grey and green border colors of bars represent *Mlkl*^fl/fl^ and *Mlkl*^LPC-KO^ groups, respectively. Error bars: means ± SEM (*p < 0.05, ***p < 0.001 and ****p < 0.0001 compared *Mlkl*^LPC-KO^ to *Mlkl*^fl/fl^ mice under similar experimental conditions; ^$^*p* < 0.05, ^$$^*p* < 0.01 and ^$$$^*p* < 0.001 compared *Mlkl*^fl/fl^ diabetic mice on HFHSD vs on SD; ^#^*p* < 0.05, ^##^*p* < 0.01 and ^###^*p* < 0.001 compared *Mlkl*^LPC-KO^ diabetic mice on HFHSD vs on SD).

Regardless of genotype, all STZ-treated mice developed pathological hyperglycemia, with fed-state blood glucose levels consistently above 250 mg/dL (normal range in healthy mice: 100–150 mg/dl), thereby confirming their diabetic status (Fig. S1). Monitoring body mass gain in *Mlkl*^fl/fl^ and *Mlkl*^LPC-KO^ mice throughout the protocol revealed similar kinetics (Fig. 1A). Similarly, hepatomegaly developed at comparable rates over time regardless of genotype (Fig. 1B). Evaluation of liver damage through serum transaminases (ALT) measurements showed normal levels in diabetic mice on standard diet (SD), although *Mlkl*^LPC-KO^ mice exhibited significantly lower ALT levels than *Mlkl*^fl/fl^ mice (64.4 IU/L versus 101 IU/L, respectively) (Fig. 1C). Their levels increased slightly in *Mlkl*^LPC-KO^ mice after 4 and 8 weeks of HFHSD, but were never significantly different from those measured in *Mlkl*^fl/fl^ mice at equivalent times. Liver damage was further assessed using TUNEL and cleaved caspase-3 staining of tissue sections at the steatosis stage (4 weeks of HFHSD), timepoint at which accumulated fatty acids led to hepatocyte death (Fig. 1D). Once more, no discernible difference was observed between the *Mlkl*^fl/fl^ and *Mlkl*^LPC-KO^ genotypes. RT-qPCR analysis of *Mlkl* expression revealed a drastic reduction of its mRNA in the liver of *Mlkl*^LPC-KO^ mice, reflecting efficient genetic deletion in liver parenchymal cells (Fig. 1E). Besides, *Mlkl*^fl/fl^ controls displayed a consistent induction at all time points of HFHSD exposure (4, 8, and 12 weeks). However, the active phosphorylated form of MLKL could not be detected by Western blot in liver extracts from *Mlkl*^fl/fl^ mice exposed to HFHSD (Fig. 1F). This lack of detectable p-MLKL did not exclude the possibility of necroptosis occurring in a limited number of hepatocytes, as HFHSD induced only a chronic mild hepatitis with low levels of cell death (Fig. 1C, D).

**Fig. 2:**
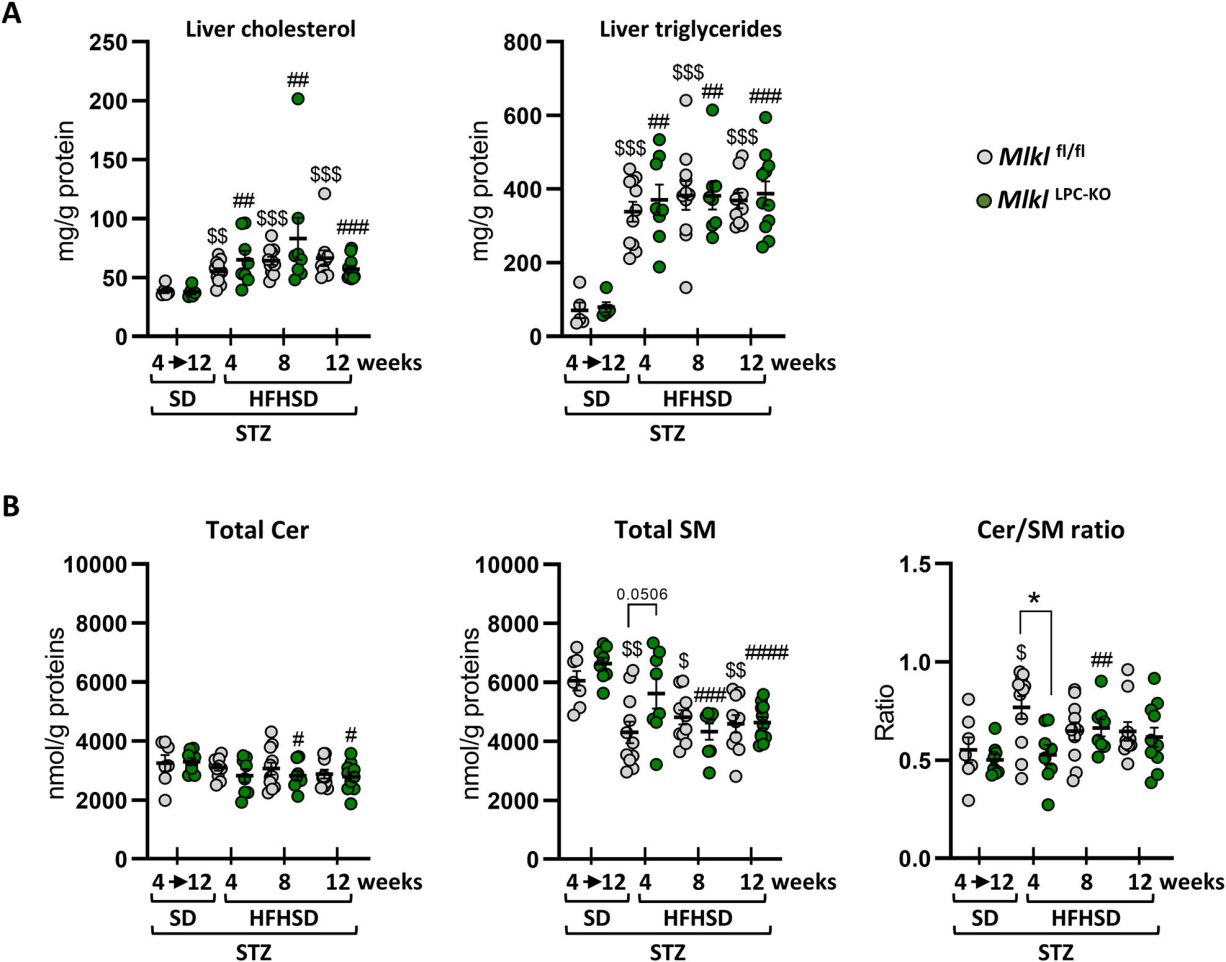
Effect of MLKL deficiency in liver parenchymal cells on hepatic lipid levels during MASLD progression. *Mlkl*^fl/fl^ and *Mlkl*^LPC-KO^ mice with streptozotocin (STZ)-induced diabetes were fed either a standard diet (SD) for a period of 4 to 12 weeks or a high-fat high-sugar diet (HFHSD) for 4, 8, or 12 weeks. **A** Hepatic cholesterol and triglyceride concentrations (mg/g protein). **B** Hepatic content of total ceramides (Total Cer, left panel), of total sphingomyelins (Total SM, middle panel) and hepatic ceramide-to-sphingomyelin ratio (Cer/SM ratio, right panel). Each grey and green dots represent *Mlkl*^fl/fl^ and *Mlkl*^LPC-KO^ individuals, respectively. Error bars: means ± SEM (*p < 0.05 compared *Mlkl*^LPC-KO^ to *Mlkl*^fl/fl^ mice under similar experimental conditions; ^$^*p* < 0.05, ^$$^*p* < 0.01 and ^$$$^*p* ^<^ 0.001 compared *Mlkl*^fl/fl^ diabetic mice on HFHSD vs on SD; ^#^*p* < 0.05, ^##^*p* < 0.01, ^###^*p* < 0.001 and ^####^*p* < 0.0001 compared *Mlkl*^LPC-KO^ diabetic mice on HFHSD vs on SD).

Liver histological analysis of *Mlkl*^fl/fl^ and *Mlkl*^LPC-KO^ mice revealed the presence of micro- and macrovesicular steatosis starting from the 4^th^ week of HFHSD (Fig. 1G). However, no discernible difference appeared between the two groups regarding NAFLD activity score (NAS) evolution (Fig. 1H). Liver fibrosis was then evaluated using Second Harmonic Generation (SHG) microscopy, a label-free technique that directly detects fibrillar collagen. The dietary regimen, known to induce mild fibrosis [24], showed no significant changes over the follow-up period for both genotypes (Fig. 1I). Gene expression analysis of fibrosis-associated markers (*Tgfβ1*, *Col1a1* and *Acta2*) corroborated the limited fibrosis development (data not shown). Quantification of hepatic cholesterol and triglyceride content confirmed the development of steatosis, with lipid accumulation detectable as early as the 4th week of HFHSD and persisting up to 12 weeks (Fig. 2A). However, no quantitative differences were observed between *Mlkl*^fl/fl^ and *Mlkl*^LPC-KO^ mice. Given their established contribution to lipotoxicity, inflammation, and fibrogenesis in MASLD progression [28], we also quantified hepatic sphingolipids and calculated the total ceramide-to-sphingomyelin (Cer/SM) ratio, a marker of metabolic stress associated with MASH (Fig. 2B). In *Mlkl*^fl/fl^ mice, this ratio was already significantly increased at 4 weeks of HFHSD, mainly driven by a reduction in total SM, whereas this decrease of SM appeared delayed in *Mlkl*^LPC-KO^ mice until 8 weeks. Species-level analysis further revealed higher Cer/SM ratios in *Mlkl*^fl/fl^ compared with *Mlkl*^LPC-KO^ mice at 4 weeks of HFHSD for 18:0, 20:0, 20:1, 22:0, 24:0, and 24:1 species (Fig. S2).

**Fig. 3:**
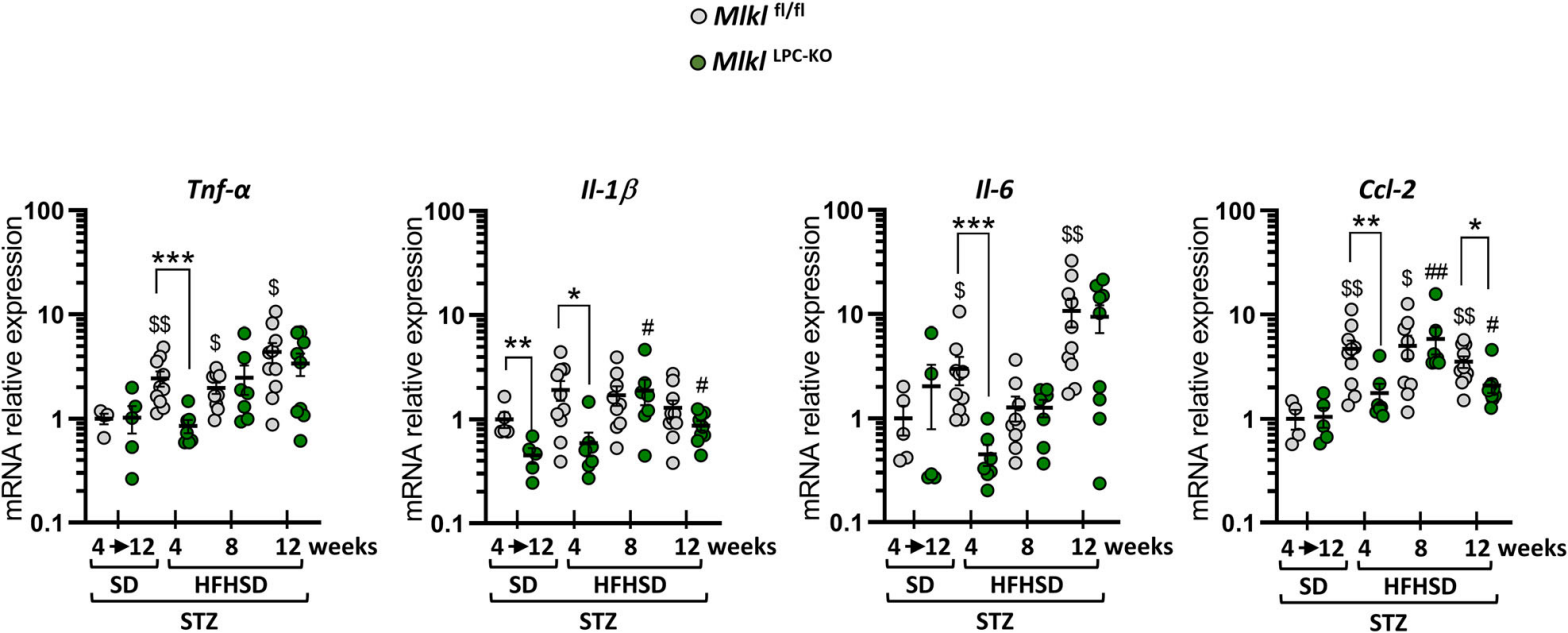
Effect of MLKL deficiency in liver parenchymal cells on the initiation of the inflammatory phase. *Mlkl*^fl/fl^ and *Mlkl*^LPC-KO^ mice with streptozotocin (STZ)-induced diabetes were fed either a standard-diet (SD) for a period of 4 to 12 weeks or a high-fat high-sugar diet (HFHSD) for 4, 8, or 12 weeks. Hepatic mRNA relative expression of *Tnf-α, Il-1β, Il-6* and *Ccl2*. Each grey and green dots represent *Mlkl*^fl/fl^ and *Mlkl*^LPC-KO^ individuals, respectively. Error bars: means ± SEM (*p < 0.05, **p < 0.01 and ***p < 0.001 compared *Mlkl*^LPC-KO^ to *Mlkl*^fl/fl^ mice under similar experimental conditions; ^$^*p* < 0.05 ^$$^*p* < 0.01 compared *Mlkl*^fl/fl^ diabetic mice on HFHSD vs on SD; ^#^*p* < 0.05 and ^##^*p* < 0.01 compared *Mlkl*^LPC-KO^ diabetic mice on HFHSD vs on SD).

Next, the hepatic inflammatory status was evaluated by measuring inflammatory gene mRNAs expression (*Tnf-α, Il-1β, Il-6* and *Ccl2*) (Fig. 3). Comparing the values between animals of the same genotype maintained on SD and those fed an HFHSD for 4 weeks revealed that 3 genes (*Tnf-α, Il-6* and *Ccl2*) showed a significant increase exclusively in *Mlkl*^fl/fl^ littermates. A similar pattern was observed for *Il-1β*. Statistically significant differences were observed between the *Mlkl*^fl/fl^ and *Mlkl*^LPC-KO^ groups at this time point for all tested genes. These differences in gene expression suggested a delay in the onset of the hepatic inflammatory phase in *Mlkl*^LPC-KO^ mice.

### MLKL lacking in LPCs postponed the splenomegaly and influenced splenic immune cell composition

**Fig. 4:**
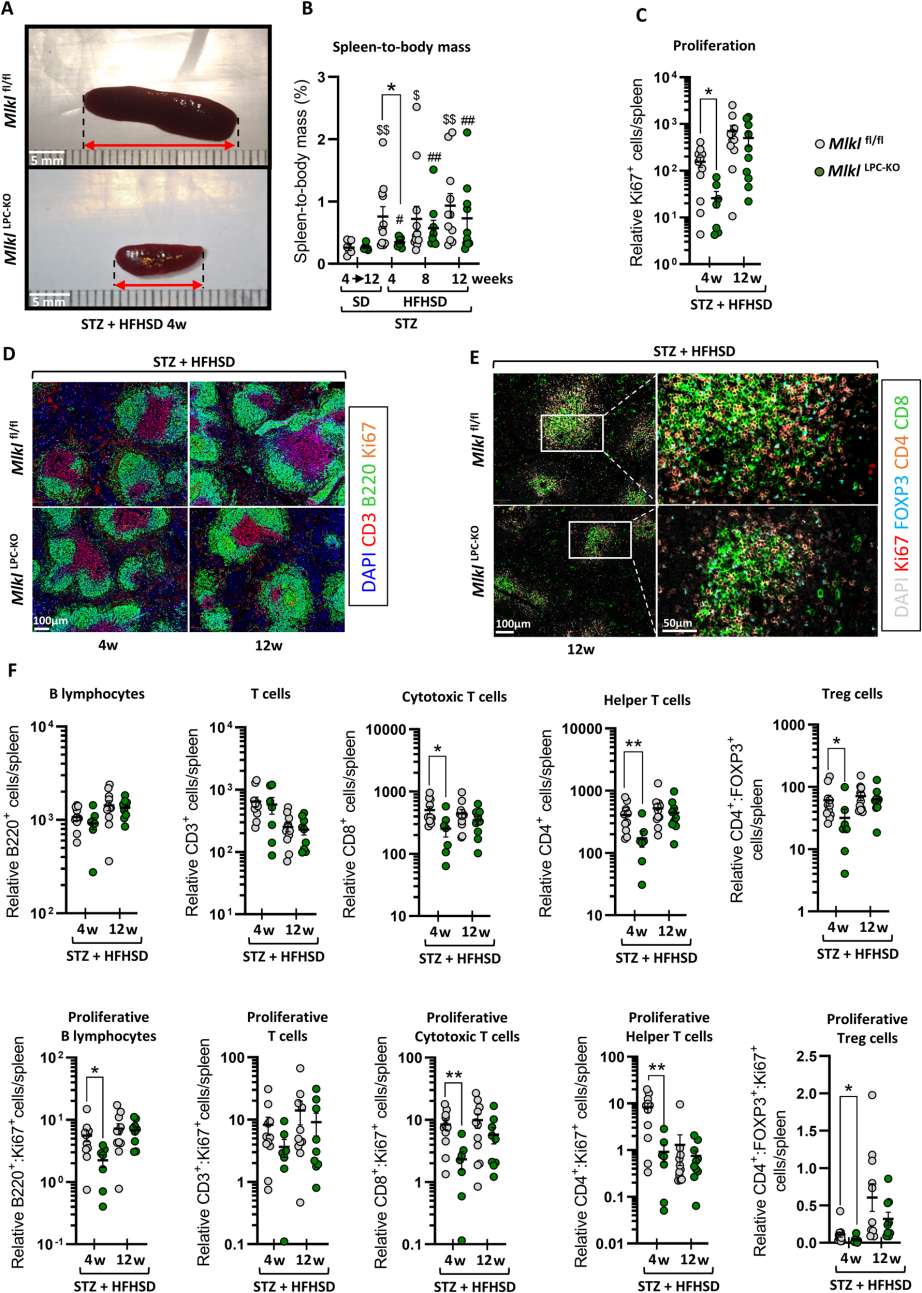
Effect of MLKL deficiency in liver parenchymal cells on splenic inflammation during MASH-HCC progression. *Mlkl*^fl/fl^ and *Mlkl*^LPC-KO^ mice with streptozotocin (STZ)-induced diabetes were fed either a standard diet (SD) for a period of 4 to 12 weeks or a high-fat high-sugar diet (HFHSD) for 4, 8, or 12 weeks. **A** Representative macroscopic images of spleens from *Mlkl*^fl/fl^ and *Mlkl*^LPC-KO^ diabetic mice under HFHSD for 4 weeks. Scale bars: 5 mm. **B** Spleen mass as % of body mass. **C** Relative quantification of proliferative cells (Ki67 + ) per spleen. **D** Representative images of CD3, B220, Ki67 and DAPI immunostaining of spleen section. Scale bars: 100 µm. **E** Representative immunostaining images of FOXP3, CD4, CD8, Ki67 and DAPI of spleen. Scale bars: 100 µm. **F** Relative quantification of B lymphocytes (B220 + ), T lymphocytes (CD3 + ), cytotoxic T cells (CD8 + ), helper T cells (CD4 + ), and regulatory T cells (CD4 + FOXP3 + ) per spleen (upper panels), and their proliferating states (Ki67 + ) (lower panels). Each grey and green dots represent *Mlkl*^fl/fl^ and *Mlkl*^LPC-KO^ individuals, respectively. Error bars: means ± SEM (*p < 0.05 and **p < 0.01 compared *Mlkl*^fl/fl^ to *Mlkl*^LPC-KO^ mice under similar experimental conditions; ^$^*p* < 0.05 and ^$$^*p* < 0^.^01 compared *Mlkl*^fl/fl^ diabetic mice on HFHSD vs on SD; ^#^*p* < 0.05 and ^##^*p* < 0.01 compared *Mlkl*^LPC-KO^ diabetic mice on HFHSD vs on SD).

After 4 weeks of HFHSD, *Mlkl*^fl/fl^ mice exhibited a larger spleen than *Mlkl*^LPC-KO^ mice (Fig. 4A). Splenomegaly developed in all animals submitted to HFHSD (Fig. 4B). This systemic effect is typically observed during MASH in patients [29] and has also been described in murine models of diet-induces MAFLD [30, 31]. In our study, this effect was more pronounced in *Mlkl*^fl/fl^ mice after 4 weeks of HFHSD. Spleen mass as % of body mass increased by approximately threefold, compared to a mere 1.3-fold increase observed in *Mlkl*^LPC-KO^ mice. Splenomegaly persisted at 8 and 12 weeks, with no significant difference between the two genotypes. Furthermore, Ki67-immunostaining at 4 and 12 weeks of HFHSD revealed increased proliferating cells in the spleen with disease progression (Fig. 4C). When organ mass was taken in consideration, the relative overall number of Ki67-positive cells within the spleen differed between the two genotypes only at the initial stage of the disease, with higher values observed in *Mlkl*^fl/fl^ mice. A multiplex immunohistochemistry approach allowed to specifically focus on splenic lymphocytes (Fig. 4D, E). When both cell density and spleen mass were considered, analysis of the relative total number of different lymphocyte subpopulations revealed no significant differences in overall B or T lymphocyte populations between the two genotypes in both early and late disease stages (Fig. 4F, top panels). However, spleens of *Mlkl*^LPC-KO^ mice showed fewer cytotoxic, helper and regulatory T cells at 4 weeks of HFHSD. Ki67 labelling revealed that, after 4 weeks on HFHSD, the spleen of *Mlkl*^fl/fl^ mice contained a greater number of dividing B cells and dividing cytotoxic, helper and regulatory T cells (Fig. 4F, bottom panels).

### The absence of MLKL in LPCs, altered the immune cells composition and the expression of the immune checkpoints PD-L1 and PD-1 in the liver

**Fig. 5:**
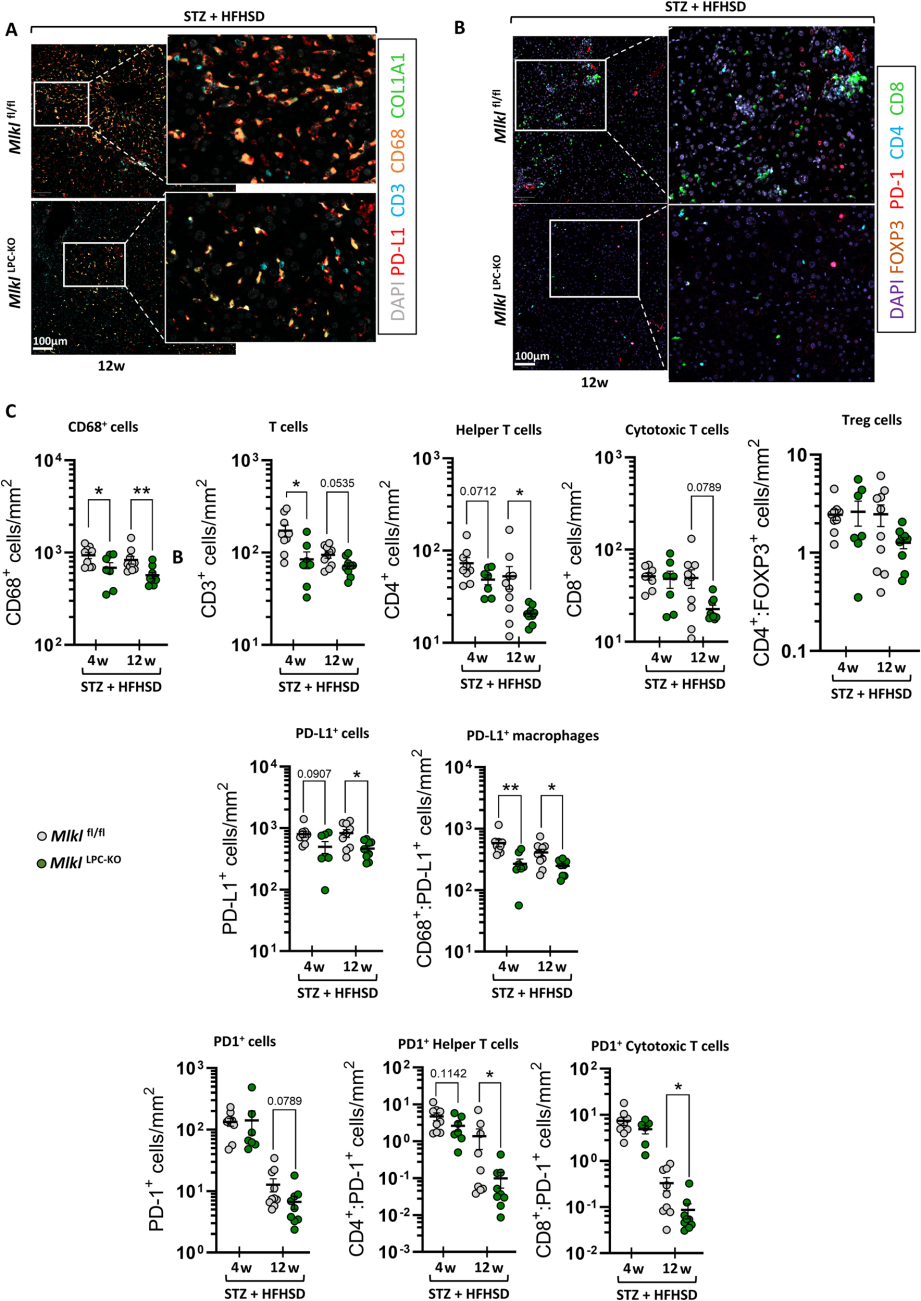
Effect of MLKL deficiency in liver parenchymal cells on liver inflammation during MASH-HCC progression. *Mlkl*^fl/fl^ and *Mlkl*^LPC-KO^ mice with streptozotocin (STZ)-induced diabetes were fed a high-fat high-sugar diet (HFHSD) for 4 or 12 weeks. **A** Representative immunostaining of PD-L1, CD3, CD68, COL1A1 and DAPI on liver sections. Scale bars: 100 µm. **B** Representative immunostaining of FOXP3, PD-1, CD4, CD8 and DAPI on liver sections. Scale bars: 100 µm. **C** Density (cells per mm²) of liver macrophages (CD68 + ), T lymphocytes (CD3 + ), helper T cells (CD4 + ), cytotoxic T cells (CD8 + ), regulatory T cells (CD4 + FOXP3 + ), PD-L1 + , CD68 + PD-L1 + , PD1 + , CD4 + PD1+ and CD8 + PD-1+ cells. Each grey and green dots represent *Mlkl*^fl/fl^ and *Mlkl*^LPC-KO^ individuals, respectively. Error bars: means ± SEM (*p < 0.05 and **p < 0.01 compared *Mlkl*^fl/fl^ to *Mlkl*^LPC-KO^ mice under similar experimental conditions).

The immune cell composition in livers and the expression of key immune checkpoints involved in HCC development were considered using multiplex immunohistochemistry (Fig. 5A, B). Results of these analysis were consistent with those previously obtained by relative quantification of inflammatory cytokine mRNAs (Fig. 3). After 4 weeks of HFHSD, *Mlkl*^fl/fl^ mice exhibited significantly higher densities of intrahepatic macrophages (CD68 + ) and T lymphocytes (CD3 + ) compared to *Mlkl*^LPC-KO^ mice (Fig. 5C, top panels). This difference in macrophage density was primarily driven by pro-inflammatory M1 macrophages, as suggested by RT-qPCR profiling showing increased M1 but not M2 markers (data not shown). No differences were observed in the subpopulations of helper (CD4 + ), cytotoxic (CD8 + ) and regulatory (CD4+ FoxP3 + ) T lymphocytes. Following a 12-week period on HFHSD, *Mlkl*^LPC-KO^ mice showed a reduced number of macrophages and helper T cells, with other cell types remaining similar between genotypes, although a downward trend was emerging.

Regarding immune checkpoints, the total number of PD-L1+ cells in the liver remained constant over time within each genotype. However, at 12 weeks of HFHSD, *Mlkl*^LPC-KO^ mice exhibited fewer PD-L1+ cells (Fig. 5C, middle panels). Besides, a lower number of PD-L1+ tumor-associated macrophages (TAMs: CD68 + PDL-1 + ) was present in the livers of *Mlkl*^LPC-KO^ mice at both 4 and 12 weeks. The number of PD-1+ cells, which was comparable between genotypes at the week 4, fell in both groups at week 12. This decline was observed in both PD-1+ helper and cytotoxic lymphocytes. However, it was significantly more pronounced in the *Mlkl*^LPC-KO^ mouse group (Fig. 5C, lower panels).

### *Mlkl*^*LPC-KO*^ mice showed a marked reduction in liver carcinogenesis, particularly in HCC incidence

**Fig. 6:**
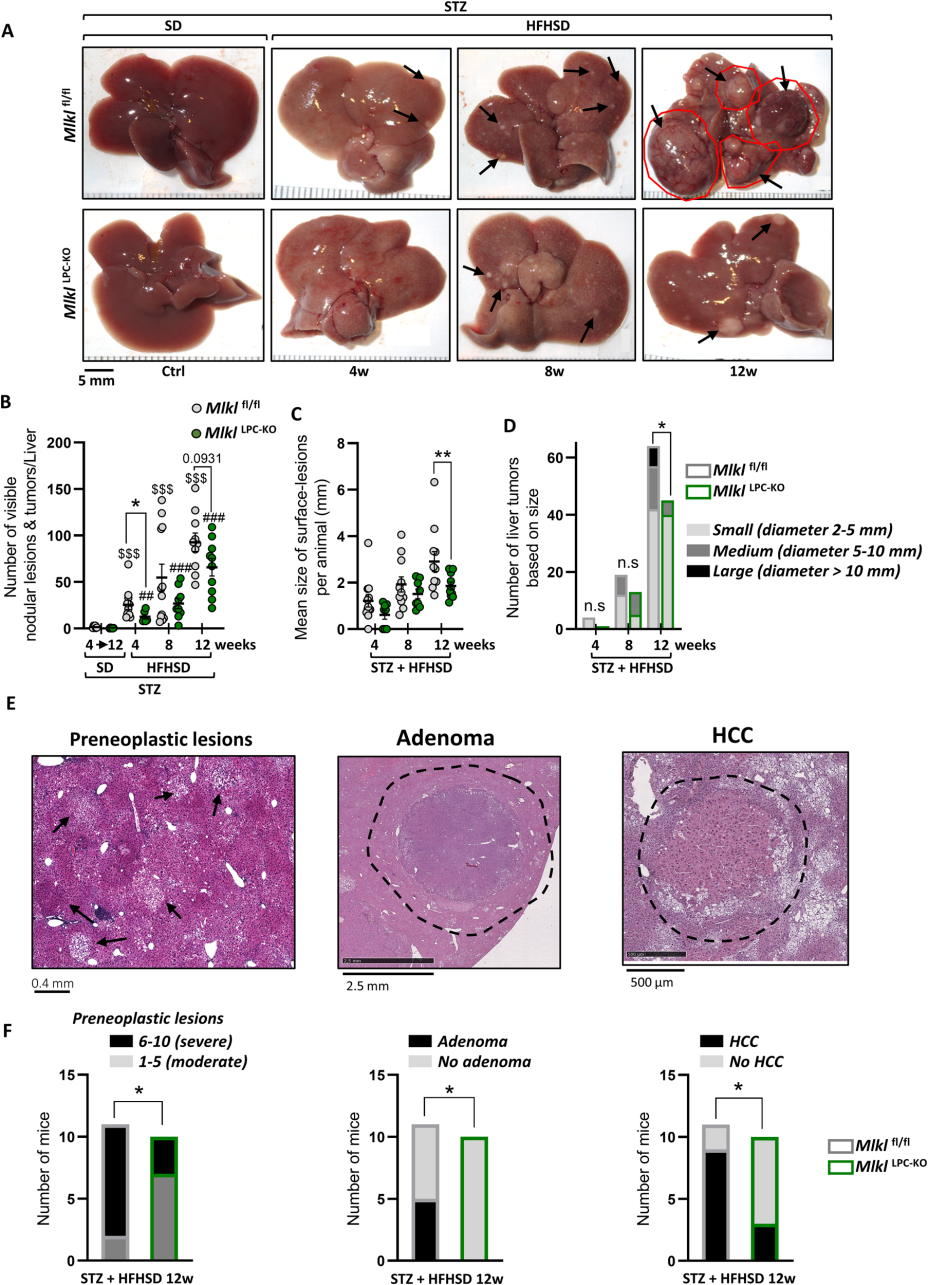
Effect of MLKL deficiency in liver parenchymal cells on liver carcinogenesis in MASH-HCC. *Mlkl*^fl/fl^ and *Mlkl*^LPC-KO^ mice with streptozotocin (STZ)-induced diabetes were fed either a standard diet (SD) for a period of 4 to 12 weeks or a high-fat high-sugar diet (HFHSD) for 4, 8, or 12 weeks. **A** Representative macroscopic images of livers from *Mlkl*^fl/fl^ (top panels) and *Mlkl*^LPC-KO^ (bottom panels) diabetic mice under SD (Ctrl) or HFHSD for indicated weeks (w). Black arrows indicate some nodular lesions and tumors. Scale bars: 5 mm. **B** Number of nodular lesions and tumors on liver surface. **C** Mean size (mm) of lesions at liver surface of each animal. **D** Tumor size distribution (small [2–5 mm], medium [5–10 mm] and large [>10 mm] respectively represented in light gray, medium gray and black); sample size (n) for each group, from left to right: 11, 8, 11, 8, 11, and 10. **E** H&E staining showing preneoplastic lesions (left panel), adenoma, (middle panel) and hepatocellular carcinoma (HCC, right panel) in liver sections from diabetic mice after 12 weeks of HFHSD. Black arrows: preneoplastic lesions; dashed black lines: adenoma or HCC boundary. Scale bars: 0.4 mm, 2.5 mm and 500 µm, respectively. **F** Graphical representations of the contingency table illustrating preneoplastic lesions according to their severity (left chart), and of adenoma (middle chart) and HCC incidence (right chart) after 12 weeks of HFHSD; sample size (*n*) for each group, from left to right: 11, and 10. For all graphs, each grey and green dots represent *Mlkl*^fl/fl^ and *Mlkl*^LPC-KO^ individuals, respectively. For all charts, grey and green border colors of bars represent *Mlkl*^fl/fl^ and *Mlkl*^LPC-KO^ groups, respectively. Error bars: means ± SEM (*p < 0.05 and **p < 0.01 compared *Mlkl*^fl/fl^ to *Mlkl*^LPC-KO^ mice under similar experimental conditions; ^$$$^*p* < 0.001 compared *Mlkl*^fl/fl^ diabetic mice on HFHSD vs on SD, or ^##^*p* < 0.01 and ^###^*p* < 0.001 compared *Mlkl*^LPC-KO^ diabetic mice on HFHSD vs on SD).

HFHSD induced liver lesions and HCC in both diabetic *Mlkl*^fl/fl^ and *Mlkl*^LPC-KO^ mice. Indeed, macroscopic examination revealed numerous nodular lesions and tumors on the liver of HFHSD-fed mice, which were absent in SD-fed mice (Fig. 6A). Interestingly, in comparison to *Mlkl*^fl/fl^ mice, *Mlkl*^LPC-KO^ mice exhibited delayed and reduced tumor development, with fewer lesions and tumors (Fig. 6B), associated with smaller tumors observed at 12 weeks (Fig. 6C). Furthermore, a classification of liver tumors according to their diameter (2-5 mm: small; 5-10 mm: medium; > 10 mm: large) unveiled a significant reduction in the number of medium tumors and the absence of large tumors in the liver of *Mlkl*^LPC-KO^ mice after 12 weeks of treatment, in contrast to what was observed in *Mlkl*^fl/fl^ mice (Fig. 6D).

The severity of preneoplastic lesions, as well as the incidence of adenomas and HCC, was determined through histopathological analysis of livers from mice that had been fed HFHSD for 12 weeks (Fig. 6E). Firstly, the classification of preneoplastic lesions according to their severity (moderate: stages 1 to 5; severe: stages 6 to 10) showed a lower incidence of the number of individuals affected by severe preneoplastic lesions in *Mlkl*^LPC-KO^ mice (Fig. 6F, left graph). Additionally, adenomas were absent in *Mlkl*^LPC-KO^ mice, while almost half of the *Mlkl*^fl/fl^ mice exhibited these benign tumors (Fig. 6F, middle graph). Finally, the incidence of HCC was found to be significantly lower in the *Mlkl*^LPC-KO^ group (Fig. 6F, right graph).

**Fig. 7:**
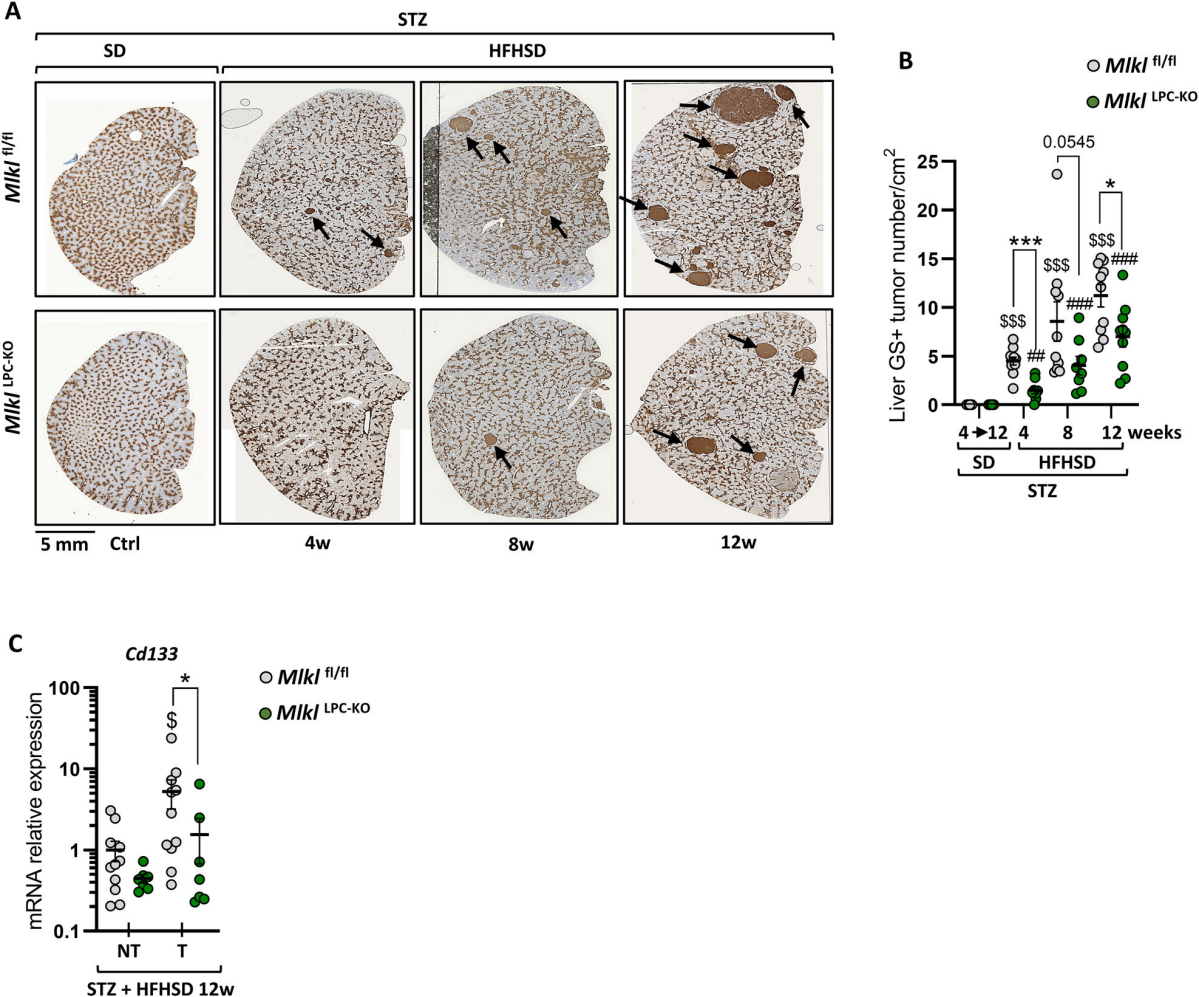
Effect of MLKL deficiency in liver parenchymal cells on HCC prevalence in MASH context. *Mlkl*^fl/fl^ and *Mlkl*^LPC-KO^ mice with streptozotocin (STZ)-induced diabetes were fed either a standard diet (SD) for a period of 4 to 12 weeks or a high-fat high-sugar diet (HFHSD) for 4, 8, or 12 weeks. **A** Representative images of glutamine synthetase (GS) staining on liver sections from *Mlkl*^fl/fl^ (top panels) and *Mlkl*^LPC-KO^ (bottom panels) diabetic mice under SD (Ctrl) or HFHSD for indicated weeks (w). Black arrows indicate GS-positive tumors. Scale bars: 5 mm. **B** Density of GS-positive (GS + ) tumors (per cm^2^ on liver section). **C** mRNA relative expression levels of *Cd133* in non-tumoral (NT) and tumoral (T) liver tissues. Each grey and green dots represent *Mlkl*^fl/fl^ and *Mlkl*^LPC-KO^ individuals, respectively. Error bars: means ± SEM (*p < 0.05 compared *Mlkl*^fl/fl^ to *Mlkl*^LPC-KO^ mice under similar experimental conditions; ^$$$^*p* < 0.001 compared *Mlkl*^fl/fl^ diabetic mice on HFHSD vs on SD, or ^##^*p* < 0.01 and ^###^*p* < 0.001 compared *Mlkl*^LPC-KO^ diabetic mice on HFHSD vs on SD); ^$^p < 0.05 compared *Mlkl*^fl/fl^ T to *Mlkl*^fl/fl^ NT.

Immunostaining of glutamine synthetase (GS), a marker used in clinical practice to diagnose some HCC, was employed as an alternative and complementary method to assess HCC development (Fig. 7A) [32, 33]. Results confirmed that diabetic mice on HFHSD developed GS+ tumors, while those fed with SD did not. Furthermore, *Mlkl*^LPC-KO^ mice on HFHSD exhibited a consistently lower incidence of GS+ tumors compared to *Mlkl*^fl/fl^ mice across all disease stages (Fig. 7B). Besides, C*d133* mRNA levels, a marker for cancer stem cell in HCC [34], were compared in non-tumor and tumor liver tissues from mice fed HFHSD for 12 weeks (Fig. 7C). Interestingly, in *Mlkl*^fl/fl^ mice, *Cd133* mRNA levels were significantly higher (5.3-fold) in tumor areas than in non-tumor areas. A similar trend was observed in *Mlkl*^LPC-KO^ mice (3.5-fold increase), although this difference did not reach statistical significance.

### Deficiency of *Mlkl* in LPCs has an impact on liver oxidative stress & DNA damage

**Fig. 8:**
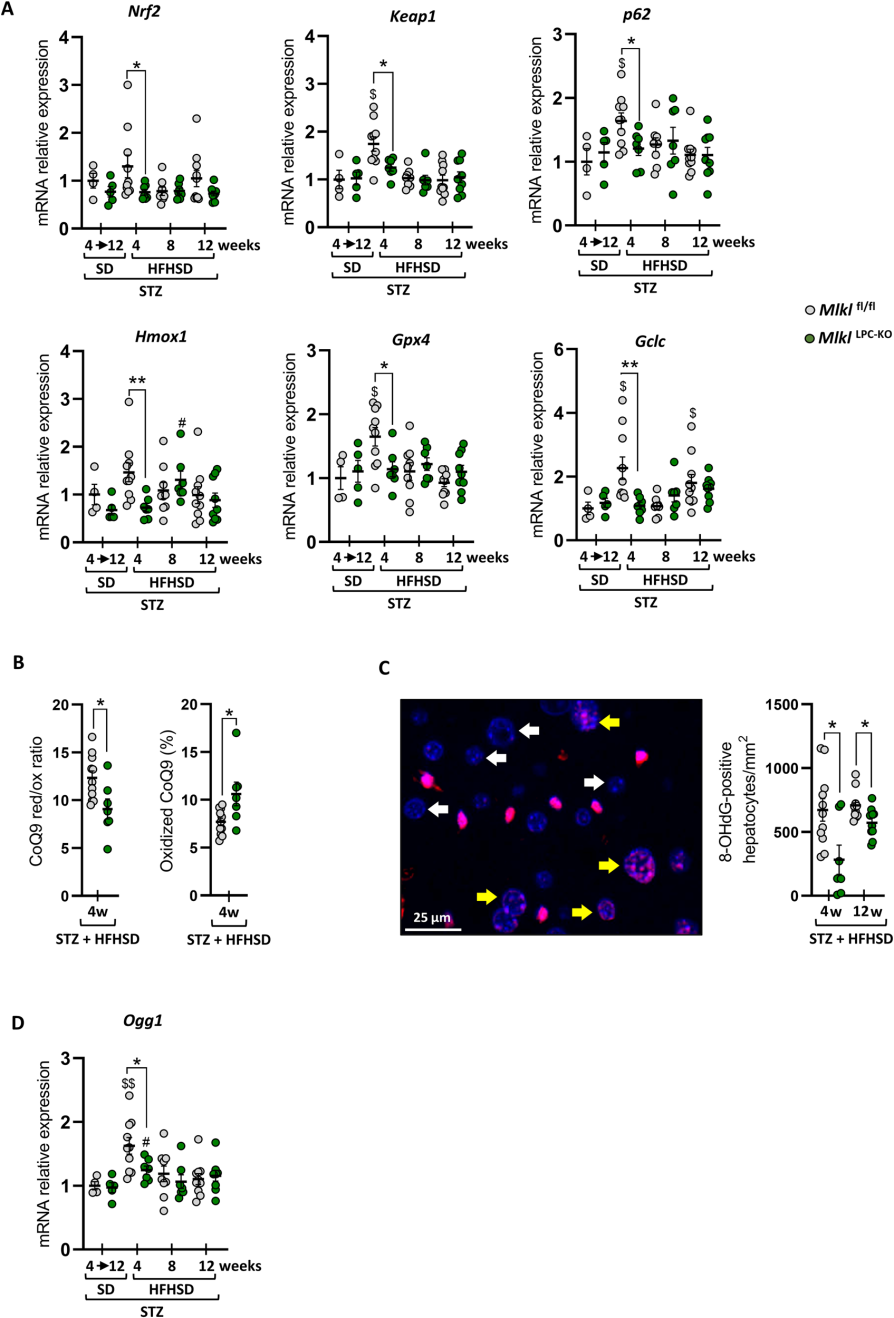
Effect of MLKL deficiency in liver parenchymal cells on oxidative stress during MASH-HCC. *Mlkl*^fl/fl^ and *Mlkl*^LPC-KO^ mice with streptozotocin (STZ)-induced diabetes were fed either a standard diet (SD) for a period of 4 to 12 weeks or a high-fat high-sugar diet (HFHSD) for 4, 8, or 12 weeks. **A** Hepatic mRNA relative expression levels of *Nrf2, Keap1, p62, Hmox1, Gpx4* and *Gclc*. **B** Reduced-to-oxidized CoQ9 ratio (left panel) and fraction of oxidized CoQ9 (right panel). **C** Representative 8-OHdG immunostaining (red) of liver section counterstained with DAPI (blue). White arrows: 8-OHdG-negative hepatocyte nuclei; yellow arrows: 8-OHdG-positive hepatocyte nuclei. Scale bars: 25 µm; density of 8-OHdG-positive hepatocytes (per mm^2^). **D** Hepatic mRNA relative expression levels of *Ogg1*. Each grey and green dots represent *Mlkl*^fl/fl^ and *Mlkl*^LPC-KO^ individuals, respectively. Error bars: means ± SEM (*p < 0.05 and **p < 0.01 compared *Mlkl*^fl/fl^ to *Mlkl*^LPC-KO^ mice under similar experimental conditions; ^$^*p* < 0.05 and ^$$^*p* < 0^.^01 compared *Mlkl*^fl/fl^ diabetic mice on HFHSD vs on SD, or ^#^*p* < 0.05 compared *Mlkl*^LPC-KO^ diabetic mice on HFHSD vs on SD).

To unravel the mechanisms underlying the observed protective effect on inflammation and HCC development in *Mlkl*^LPC-KO^ mice, oxidative stress and DNA damage were investigated in the liver. A comparison of the mRNA expression levels of oxidative stress-induced genes (*Nrf2, Keap1, p62, Hmox1, Gpx4* and *Gclc*) revealed significant lower levels in the liver of *Mlkl*^LPC-KO^ mice at the 4-week stage (Fig. 8A). To further investigate the origin of oxidative stress in the liver of mice fed HFHSD for 4 weeks, we assessed potential mitochondrial contribution, as mitochondria represent a major source of reactive oxygen species (ROS) in hepatocytes. To this end, we measured the hepatic CoQ9 redox ratio (CoQ9H₂/CoQ9), which was slightly but significantly higher in *Mlkl*^fl/fl^ mice compared to *Mlkl*^LPC-KO^ mice (Fig. 8B). A recent study demonstrated that an increase in this ratio under steatotic conditions promotes mitochondrial ROS production through reverse electron transport (RET) [35]. Consistently, *Mlkl*^LPC-KO^ mice displayed a significantly higher oxidized-to-total CoQ9 pool, reflecting a more oxidized state less prone to ROS generation via RET. Next, the presence of oxidative DNA damage was determined through the utilization of 8-hydroxydeoxyguanosine (8-OHdG) staining on liver sections following 4 or 12 weeks of HFHSD (Fig. 8C) [36]. The density of hepatocytes with 8-OHdG positive nuclear staining was found to be systematically significantly more elevated in the *Mlkl*^fl/fl^ mice, with a more pronounced discrepancy observed at the 4-week stage. To complete these data, the mRNA levels of *Ogg1*, a gene encoding an enzyme involved in DNA damage repair and 8-OHdG removal [37, 38], were compared between genotypes (Fig. 8D). Regardless of genotype, this gene was induced at the fourth week of HFHSD and subsequently returned to its baseline level at later times. Nevertheless, the induction was found to be significantly greater in *Mlkl*^fl/fl^ mice.

## Discussion

The occurrence of necroptosis in liver diseases remains a subject of controversy. Notably, a recent study, focused on acute and chronic hepatitis, including MASH induced by a high-fat diet (HFD), has reported that the global knockout of MLKL (*Mlkl*^-/-^) did not offer protection to mice against these pathologies [39]. They attributed these findings to the potential inability of hepatocytes to undergo necroptosis, due to the lack of RIPK3 expression in these cells. However, other studies have demonstrated that the active phosphorylated form of MLKL (pMLKL) was present in the hepatocyte plasma membrane of mice treated with CCl_4_, as well as in both mouse and human MASH samples [40, 41]. Consistently, in Ripk3-deficient mice, diet-induced MASH failed to trigger pMLKL, a phenotype that has been associated with protection from disease, including reduced inflammation, fibrosis, and carcinogenesis [42]. Moreover, recent works have demonstrated that hepatocytes, despite their low endogenous RIPK3 expression, are functionally capable of undergoing necroptosis [43]. Specifically, following genetic deletions that trigger both necrosome and NF-κB activation, hepatocytes can enter a prolonged sublethal necroptotic state. In this condition, cells develop leaky membranes and act as secretory cells, releasing alarmin, which, in turn, fuel liver inflammation and promote liver cancer. Finally, it has been reported that MLKL can be activated by mechanisms other than the classical RIPK3-dependent pathway, such as cleavage by caspases [44].

Although some studies have explored MLKL’s role in MASH, none have investigated its potential involvement, particularly in LPCs, in the progression to liver cancer. To address this issue, we conducted experiments on diabetic male mice lacking *Mlkl* globally (*Mlkl*^-/-^) or only in LPCs (*Mlkl*^LPC-KO^), in parallel with their respective littermate controls (*Mlkl*^+/+^ and *Mlkl*^fl/fl^). All mice were fed an HFHSD to model the progression of the human disease to liver cancer, as previously described [24]. In our investigations, *Mlkl*^-/-^ mice exhibited no substantial disparities from *Mlkl*^+/+^ controls in any of the clinical signs assessed throughout the protocol (data not shown). Disease progression followed a similar course in both genotypes, from steatosis onset to tumor development, in contrast to the phenotypic differences observed between *Mlkl*^fl/fl^ and *Mlkl*^LPC-KO^ mice. This discrepancy could be attributed to the multifaceted roles of MLKL across diverse cell types and biological functions [12], which could interact and influence the complex processes underlying MASH-HCC pathogenesis. Thus, in macrophages, MLKL would contribute to the activation of their phagocytic function, which is essential for eliminating bacterial products leaking from the gut during MASLD in order to reduce inflammation that contributes to liver damage [45]. Consequently, in *Mlkl*^-/-^ mice, the prevention of this phagocytosis would result in the promotion of inflammation and thus counteract the protective effect observed in *Mlkl*^LPC-KO^ mice linked to the lack of MLKL in LPCs. Besides, it is noteworthy that a previous study found that male *Mlkl*^-/-^ mice had reduced inflammation and HCC incidence compared to their *Mlkl*^+/+^ counterparts, when fed a choline-deficient amino acid-defined high-fat diet (CDA-HFD) [46]. The disparities in disease progression observed across different experimental models using *Mlkl*^-/-^ mice may be attributed to various factors, including genetic background, gut microbiota and dietary composition. Specifically, diet plays a crucial role in liver inflammation and, consequently, in HCC development [24]. Notably, CDA-HFD is known to induce accelerated MASH with severe fibrosis, without causing obesity or metabolic syndrome [47]. Nevertheless, as specified above, the observed phenotype in *Mlkl*^-/-^ strain likely resulted from a combination of various impacts associated with the pseudokinase’s multifunctional nature, which vary depending on the cell type [12]. Consequently, *Mlkl* silencing specifically in LPCs of *Mlkl*^-/-^ mice may play a dominant role in pathogenesis when exposed to CDA-HFD, potentially explaining the partial protection observed [46], in contrast to the absence of phenotype detected in our study using HFHSD.

Comparing the progression of MASLD to HCC development in *Mlkl*^LPC-KO^ mice versus *Mlkl*^fl/fl^ control mice revealed a delayed onset of certain aspects of the pathological phenotype. Accordingly, the inflammatory phase was postponed, as evidenced by targeted gene expression analysis and characterization of major immune cell populations in the spleen and liver. Similarly, monitoring the emergence of pre-neoplastic lesions and their progression into adenomas and, ultimately, malignant tumors demonstrated that tumorigenesis was also delayed. Analysis of multiple tumorigenesis biomarkers supports this observation. Notably, a lower intrahepatic density of macrophages (CD68⁺ cells) was observed, together with reduced expression of macrophage-derived cytokine genes such as *Tnf-α, Il-1β*, and *Il-6*, which are known to directly target hepatocytes and promote steatosis, inflammation, and hepatocellular damage [48]. By slowing the recurrence of hepatocyte death and compensatory proliferation, key drivers of genomic instability [49, 50], the onset of HCC is consequently deferred. Furthermore, in *Mlkl*^LPC-KO^ mice, immune cells associated with impaired anti-tumor surveillance, such as CD8⁺PD-1⁺ and CD4⁺PD-1⁺ T cells [51, 52], as well as pro-angiogenic tumor-associated macrophages (TAMs: CD68⁺PD-L1⁺) [53, 54], appeared later in disease progression.

In an attempt to define the early stage of the disease at which the absence of MLKL in LPCs may impact MASH-HCC pathogenesis, intrahepatic oxidative stress levels were investigated. A comprehensive analysis of hepatic oxidative stress signatures utilizing diverse methodologies has been conducted, reflecting the presence of ROS in the livers of mice, particularly after 4 weeks of HFHSD feeding, though with considerably lower levels of stress observed in *Mlkl*^LPC-KO^ mice. These findings demonstrated that the absence of MLKL in LPCs was associated with a reduction of hepatic oxidative stress, which has been identified as a pivotal factor in MASH-HCC pathogenesis [1, 5]. Consistently, hepatic sphingolipid analysis showed that a decline in SM occurred later in *Mlkl*^LPC-KO^ mice (week 8) compared with *Mlkl*^fl/fl^ mice (week 4). SM reduction probably resulted from their hydrolysis by sphingomyelinases, particularly nSMase2 encoded by *Smpd3*, which has been reported to be induced during MASH and to contribute to inflammation and fibrosis [55, 56]. Cer derived from SM hydrolysis probably contribute in turn to inflammation through their downstream catabolism. Although we did not detect increased *Smpd3* mRNA expression in our HFHSD model (data not shown), nSMase2 activity can be directly enhanced by various stress signals, including cytokines (TNF-α, IL-1β, IFN-γ) but also ROS [57]. Thus, the delayed hydrolysis of SM in *Mlkl*^LPC-KO^ mice may result from a postponed onset of inflammation and oxidative stress linked to the lack of MLKL in LPC. In line with this, our findings suggest that MLKL played a role in shaping hepatic mitochondrial redox homeostasis in our MASH-HCC model by contributing to an altered CoQ9 redox balance that favored the accumulation of reduced CoQ9 (CoQ9H₂). Thus, MLKL may have promoted mitochondrial ROS production through RET [35]. Consistently, the more oxidized CoQ9 pool found in *Mlkl*^LPC-KO^ mice pointed to a protective effect against this ROS-producing mechanism. Interestingly, emerging evidence indicates that MLKL exerts multiple direct effects on mitochondria beyond its canonical role in necroptosis [58]. Its accumulation within mitochondria destabilizes cardiolipin-rich membranes and promotes ROS generation. Upon activation, MLKL interacts with the mitochondrial phosphatase PGAM5, which in turn activates Drp1 to induce mitochondrial fission, and phosphorylates cyclophilin D to promote mitochondrial permeability transition pore (MPTP) opening, thereby facilitating ROS release. In addition, MLKL has been shown to inhibit mitophagy, leading to the persistence of damaged mitochondria and aggravating mitochondrial dysfunction. Therefore, it can be postulated that some or all of these specific functions of MLKL on mitochondria would contribute to the reduction of oxidative stress observed in the initial pathogenesis phase in the *Mlkl*^LPC-KO^ mouse group. This would subsequently limit the onset of intrahepatic inflammation and reduce DNA damage, thereby delaying HCC progression in *Mlkl*^LPC-KO^ mice. Building on our findings, further investigations would be valuable to deepen the understanding of how MLKL-mediated mitochondrial alterations contribute to HCC development during MASH.

Future investigations including an HFHSD-only group would also help clarify whether the observed MLKL-dependent effects on oxidative stress, inflammation, and HCC development are primarily driven by lipotoxicity, hyperglycemia, or synergistic mechanisms. Nevertheless, the newly acquired data provide valuable insights into the therapeutic benefits of targeting MLKL. Indeed, for the first time, they demonstrated that its specific absence in LPCs protects against MASH-related HCC, presumably by interfering with the delivery of ROS from mitochondria in hepatocytes at early stages of the disease. This interpretation is consistent with multiple reports demonstrating that improving hepatocyte mitochondrial function reduces oxidative stress and markedly limits MASH progression and HCC development [59–61]. This study also underscores the necessity of contemplating the potential hazards associated with targeting a protein with such multifaceted functions, as the absence of MLKL in other cell types may thwart the protective effects obtained when targeted in LPCs.

## Supplementary information

Original Data

MLKL in liver parenchymal cells promotes liver cancer in murine metabolic dysfunction-associated steatotic liver disease

## Data Availability

All data generated or analyzed during this study are included in this article and its supplementary material. Further enquiries can be directed to the corresponding authors.
