# Supplementary figures and images for: MLKL in liver parenchymal cells promotes liver cancer in murine metabolic dysfunction-associated steatotic liver disease
Source: Cell Death Dis. 2026 Feb 19;17(1):229. doi: 10.1038/s41419-026-08458-x (PMC12920736; doi:10.1038/s41419-026-08458-x)

Uncropped Western Blots (corresponding to Fig. 1F).

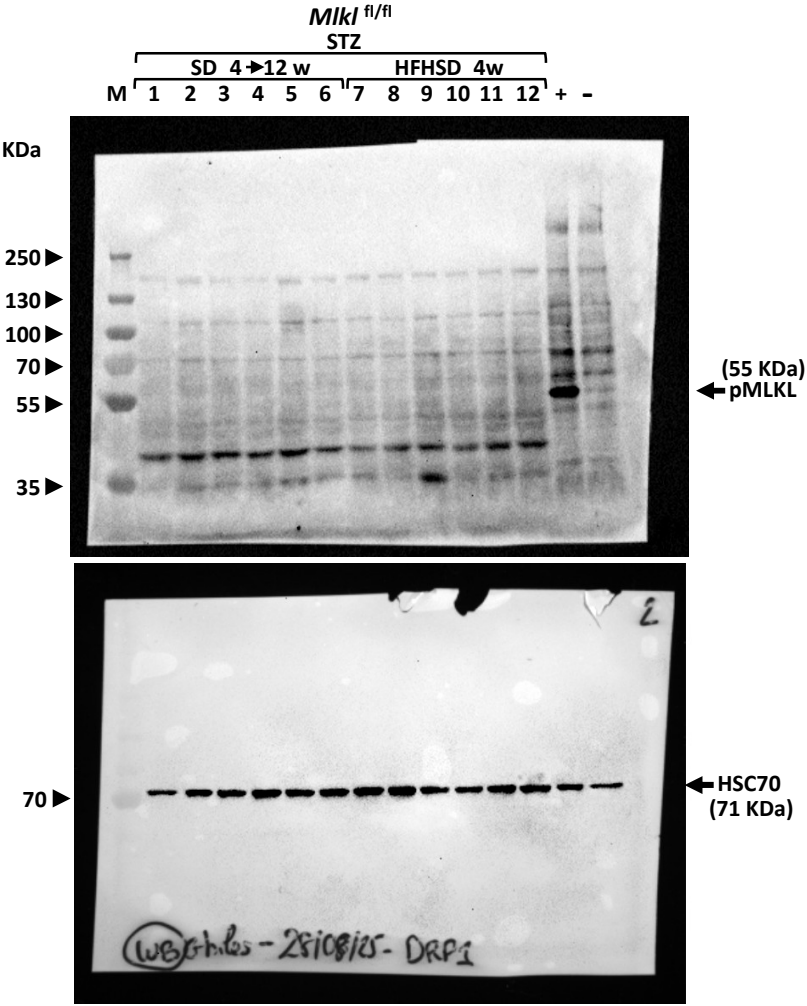

Supplement: Supplementary file 1 — Original Data [file 41419_2026_8458_MOESM1_ESM.pdf]
