# Supplementary material for: MLKL in liver parenchymal cells promotes liver cancer in murine metabolic dysfunction-associated steatotic liver disease
Source: Cell Death Dis. 2026 Feb 19;17(1):229. doi: 10.1038/s41419-026-08458-x (PMC12920736; doi:10.1038/s41419-026-08458-x)
Supplement: Supplementary file 2 — MLKL in liver parenchymal cells promotes liver cancer in murine metabolic dysfunction-associated steatotic liver disease [file 41419_2026_8458_MOESM2_ESM.docx]

***MLKL in liver parenchymal cells promotes liver cancer in murine metabolic dysfunction-associated steatotic liver disease***

Ghiles Imerzoukene, Ghania Hounana Kara-Ali, Céline Heitz-Marchaland, Thibaut Larcher, Mélanie Simoes Eugénio, Annaïg Hamon, Aurore Bidon, Gevorg Ghukasyan, Laurence Dubreil, Nicolas Loiseau, Sarah Dion, Céline Raguenes-Nicol, Claire Piquet-Pellorce, Michel Samson, Marie-Thérèse Dimanche-Boitrel and Jacques Le Seyec

**Table of contents:**

**Fig. S1:** Pathological hyperglycemia in STZ-treated mice.

**Fig. S2**: Metabolic stress in mice subjected to the MASH-HCC protocol.

**Table S1**: Sequences of primers used for real-time quantitative PCR.

**Table S2**: List of used antibodies.

**
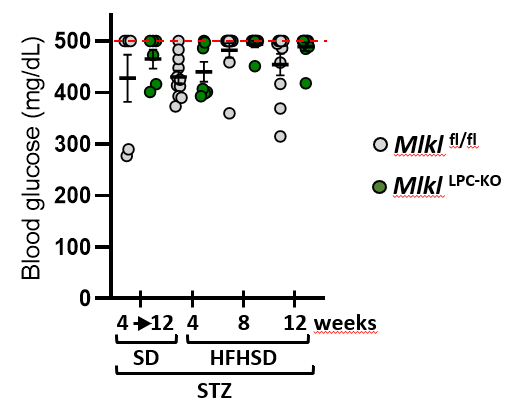
**

**Fig. S1. Pathological hyperglycemia in STZ-treated mice.** *Mlkl*^fl/fl^ and *Mlkl*^LPC-KO^ mice with streptozotocin (STZ)-induced diabetes were fed either a standard diet (SD) for a period of 4 to 12 weeks or a high-fat high-sugar diet (HFHSD) for 4, 8, or 12 weeks. Fed-state blood glucose was measure at euthanasia. Each grey and green dots represent *Mlkl*^fl/fl^ and *Mlkl*^LPC-KO^ individuals, respectively. Errors bars: means ± SEM. The glucometer used for blood glucose measurements had an upper detection limit of 500 mg/dL, indicated in the graph by a red dotted line.

**
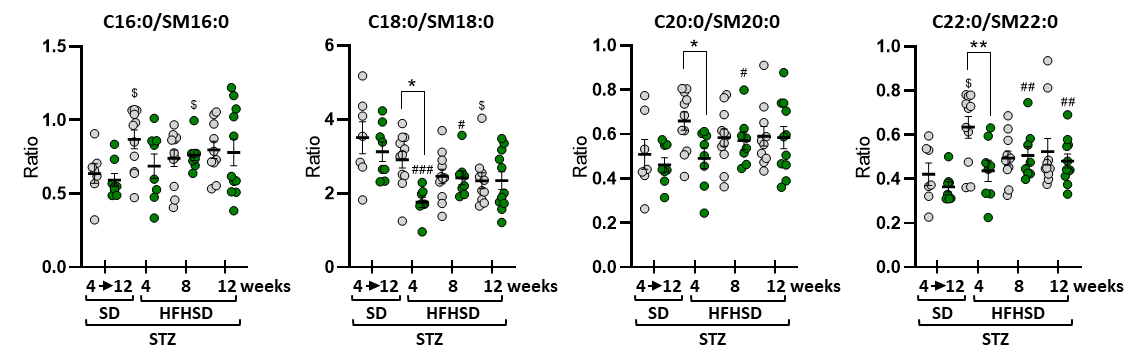

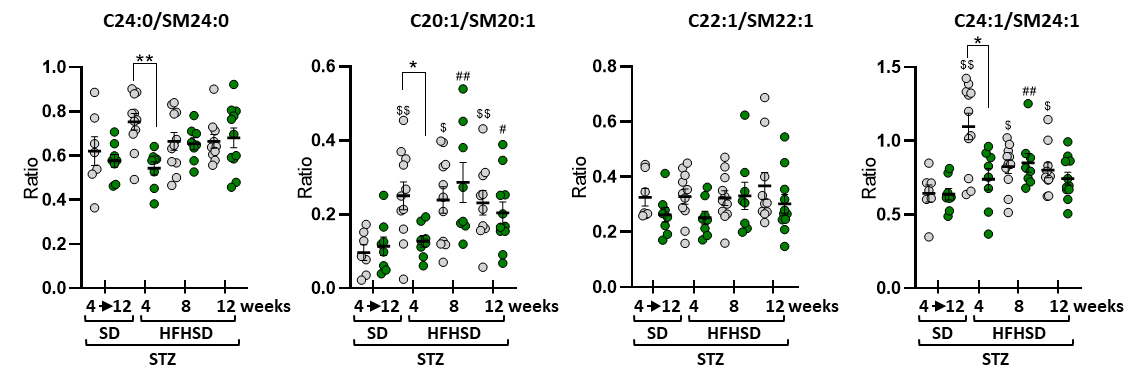

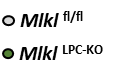
**

**Fig. S2. Metabolic stress in mice subjected to the MASH-HCC protocol.** *Mlkl*^fl/fl^ and *Mlkl*^LPC-KO^ mice with streptozotocin (STZ)-induced diabetes were fed either a standard diet (SD) for a period of 4 to 12 weeks or a high-fat high-sugar diet (HFHSD) for 4, 8, or 12 weeks. Hepatic ceramide-to-sphingomyelin ratios for individual lipid species. Each grey and green dots represent *Mlkl*^fl/fl^ and *Mlkl*^LPC-KO^ individuals, respectively. Errors bars: means ± SEM (**p* < 0.05 and ***p* < 0.01 compared *Mlkl*^LPC-KO^ to *Mlkl*^fl/fl^ mice under similar experimental conditions; ^$^*p* < 0.05 and ^$$^*p* < 0.01 compared *Mlkl*^fl/fl^ diabetic mice on HFHSD vs on SD; ^#^*p* < 0.05, ^##^*p* < 0.01 and ^###^*p* < 0.001 compared *Mlkl*^LPC-KO^ diabetic mice on HFHSD vs on SD).

**Table. S1. Sequences of primers used for real-time quantitative PCR.**

| **Gene** | **Forward primer** | **Reverse primer** |
| --- | --- | --- |
| ***18S*** | GTAACCCGTTGAACCCCATT | CCATCCAATCGGTAGTAGCG |
| ***Mlkl*** | ATGCCAGCGTCTAGGAAACC | TGTCATCGGGCAGGTTCTTC |
| ***Tgfb1*** | CACCATCCATGACATGAACC | CAGAAGTTGGCATGGTAGCC |
| ***ColA1A*** | GAAGCACGTCTGGTTTGGA | ACTCGAACGGGAATCCATC |
| ***Acta2*** | GTCCCAGACATCAGGGAGTAA | TCGGATACTTCAGCGTCAGGA |
| ***Tnf-a*** | TAGCTCCCAGAAAAGCAAGC | TTTTCTGGAGGGAGATGTGG |
| ***Il-1b*** | GATCCACACTCTCCAGCTGCA | CAACCAACAAGTGATATTCTCCA |
| ***Il-6*** | CCGGAGAGGAGACTTCACAG | TCCACGATTTCCCAGAGAAC |
| ***Ccl2*** | TCTGGACCCATTCCTTCTTG | AGGTCCCTGTCATGCTTCTG |
| ***Cd133*** | TGGAGCTACCTGCGGTTTAGA | GGACCTGTGATTGCGATAATGA |
| ***Nrf2*** | TAGATGACCATGAGTCGCTTGC | GCCAAACTTGCTCCATGTCC |
| ***Keap-1*** | CATCCACCCTAAGGTCATGGA | GACAGGTTGAAGAACTCCTCC |
| ***p62*** | CAGGCACCCCGAAACATG | ACTTATAGCGAGTTCCCACCA |
| ***Hmox1*** | AGGTACACATCCAAGCCGAGA | CATCACCAGCTTAAAGCCTTCT |
| ***Gpx4*** | CCGGCTACAACGTCAAGTTT | TCCATTTCCACAGTGGGTGG |
| ***Gclc*** | GTGCTCAAGTGGGGTGACGA | GGGTCGGATGGTTGGGGTTT |
| ***Ogg1*** | TGAGCTGCGTCTGGACTTGGTT | CTCCGTCTGAGTCAGTGTCCA |

**Table. S2. List of used antibodies.**

| **Antibody** | **Reference** | **Tissue** | **IHC**  **(dilution)** | **IF**  **(dilution)** |
| --- | --- | --- | --- | --- |
| **Glutamine synthetase** | Abcam, ab73599 | Liver | 1/100 |  |
| **CD68** | Abcam, ab125212 | Liver |  | 1/200 |
| **B220** | BioLegend, # 553084 | Spleen |  | 1/2000 |
| **CD3** | Dako, A0452 | Liver |  | 1/1000 |
|  |  | Spleen |  |  |
| **CD8** | Abcam, ab209775 | Liver |  | 1/500 |
|  |  | Spleen |  |  |
| **CD4** | Interchim, #50134-R001 | Liver |  | 1/200 |
|  |  | Spleen |  |  |
| **FOXP3** | Fisher Scientific, #15247457 | Liver |  | 1/50 |
|  |  | Spleen |  |  |
| **PD-L1** | Cell Signaling, #13684 | Liver |  | 1/50 |
| **PD-1** | NovusBio, #NBP1-77276 | Liver |  | 1/1000 |
| **Collagen I a1 (Col1a1)** | Abcam, ab34710 | Liver |  | 1/100 |
| **8-OHdG** | Abcam, ab48508 | Liver |  | 1/100 |
| **Ki67** | Novusbio, NB600-1252 | Liver |  | 1/100 |

IHC: Immunohistochemistry; IF: Immunofluorescence
